# The Supply of Rheumatology Specialist Care in Real Life. Results of a Nationwide Survey and Analysis of Supply and Needs

**DOI:** 10.3389/fmed.2020.00016

**Published:** 2020-01-30

**Authors:** Rudolf Puchner, Anna Vavrovsky, Herwig Pieringer, Ronald Hochreiter, Klaus P. Machold

**Affiliations:** ^1^Rheumatology Practise, Wels, Austria; ^2^Academy for Value in Health GmbH, Vienna, Austria; ^3^Diakonissen Hospital Linz, Linz, Austria; ^4^Department of Finance, Accounting and Statistics, Institute for Statistics and Mathematics, Vienna University of Economics and Business, Vienna, Austria; ^5^Division of Rheumatology, Department of Internal Medicine III, Medical University of Vienna, Vienna, Austria

**Keywords:** musculoskeletal disorders, rheumatoid arthritis, rheumatology workforce, epidemiology, health services research, rheumatology care

## Abstract

**Objectives:** To study the balance between the supply and need for rheumatology care in Austria. In addition, to investigate rheumatologists' work-hours, the amount of time rheumatologists dedicate to care for patients with rheumatic and musculoskeletal diseases (RMD), with non-RMD problems, and other professional activities such as research, teaching, and administration.

**Methods:** A questionnaire covering aspects of professional activities was sent to all 215 rheumatologists registered with the Austrian Medical Association. The data collected was set in relation to the need calculated on the basis of recommendations put forward by the German society of rheumatology.

**Results:** 149 of the 215 rheumatologists (69.0%) responded. Median weekly working time was 50 h (IQR 45–60). 47.4% of the working time was spent for care of patients with RMD. The remaining time was dedicated to patients with non-rheumatic diseases (19.6%), research and teaching (8.4%), and administration (24.5%). The number of full-time equivalents (FTE, based on a 40-h work-week) available for rheumatology care, thus, was calculated to be 178.5. Based on disease prevalence/incidence estimates and on the time allocation results of this survey, our study resulted in a need of 4.29 rheumatologists per 100.000 adult inhabitants (301.79 for an adult population of 7.03 × 10^6^).

**Conclusion:** The study demonstrated a substantial mismatch between the available supply and the need for rheumatology care. The results of our study are a conservative estimate, which should be taken into consideration for future healthcare workforce planning. In particular, the rising need for rheumatologists should be met by increasing the numbers of those specialists.

## Background

In a report from 2012, the European Commission foresaw a substantial “gap in supply of human resources in health by 2020”^1^. Several reports, in various European countries, have tried to estimate the number of available rheumatologists (1). Due to the aging population, the prevalence of rheumatic musculoskeletal diseases (RMD), be they inflammatory or non-inflammatory, in the general population is expected to increase substantially. In addition, in the past decade, highly effective drugs have been developed for the treatment of inflammatory rheumatic diseases, which call for expert knowledge in their prescription and monitoring of therapy. The implementation of early referrals to specialists and the “treat to target” strategy, which aims to achieve remission or at least low disease activity, require a more stringent surveillance of patients with inflammatory rheumatic diseases (1–4).

Furthermore, due to increasing age and improved survival, both in the general population and in patients with many, not only inflammatory but also chronic diseases, the number of patients with co-morbidities is also rising (5, 6). This leads to a dramatic increase of specialist visits needed to care for patients with RMD.

Therefore, the need for well-trained specialists in rheumatology, also with a sufficiently broad background in Internal Medicine, is likely to rise continuously. This contrasts with the perceived scarcity of physicians having chosen or choosing a career in rheumatology. However, the current supply of rheumatologists and, in particular, how much time these specialists are able to provide care for patients with rheumatic problems, has not been studied in detail. Currently available data has relied mostly on “head counts” of rheumatologists in various countries (1). These head counts neglect (i) various work-time models (e.g., part time employment), and (ii) the fact that frequently, only part of the work-time is dedicated to rheumatology, as a substantial amount of time from each individual rheumatologist is required for care of patients with non-rheumatic diseases and other professional functions, such as research, teaching, and administration.

Recently Dejaco et al. announced the first EULAR endorsed “points to consider,” providing a framework for the implementation of future workforce requirement studies in rheumatology. That article highlights areas of uncertainty and indicates aspects that require future research (7). Rheumatology specialist care in Austria is, like in many other countries, mainly provided in the framework of clinics associated with hospitals and, especially in the major cities, in special clinics run by the public insurance companies. Rheumatologists are trained specialists in Internal Medicine with an additional training in rheumatology. Therefore, most Rheumatology Departments are associated with Internal Medicine Departments and have to provide care for patients with non-rheumatic (internal) diseases as well, at least in their respective inpatient facilities. The majority of rheumatologists are associated with, or employed by a hospital department. A certain percentage of these specialists also provide rheumatology care in a private practice. A minority of rheumatologists provide rheumatology care in an office setting only. Of those, only very few have a contract with public insurance, rather, they charge for their services on an individual basis (“private honorarium”).

In order to inform institutions dealing with health care planning, and to provide objective data on the amount of rheumatology services in terms of “rheumatology time” available, a survey was undertaken among all registered specialists in Internal Medicine with a sub specialization in rheumatology, in Austria. The objective of the present study was to investigate (i) the amount of time rheumatologists (in particular, specialists in Internal Medicine and rheumatology) dedicate to care for patients with RMD (ii) the amount of time rheumatologists dedicate to care of patients with inflammatory vs. non-inflammatory RMDs, (iii) the amount of time rheumatologists dedicate to care of patients with non-RMD problems, and (iv) the proportion of work time spent on other professional activities such as research, teaching and administration. Furthermore, we are attempting to investigate the balance between the available supply and the need for rheumatology care and, thus, the number of rheumatologists necessary to satisfy this need in Austria.

## Methods

### Participants

Because it is mandatory to be registered with the Austrian Medical Association to practice medicine, a complete list of all specialists in Internal Medicine and rheumatology was available. These were invited to participate in the study, regardless of whether they were actively involved in care of RMD patients or not (“title only” rheumatologists). The survey questionnaire could either be taken online or by telephone interview (whichever the respondent preferred). Participants were invited by e-mail or telephone, or both, and reminded three times (by telephone) to complete the questionnaire. The structure and questions were identical in the online questionnaire and in the telephone interview. The telephone interview was taken by trained personnel, provided by the Academy for Value in Health.

### Questionnaire

The questionnaire covered the following aspects of working-time distribution: weekly working hours as a physician; divided in weekly working hours dedicated to rheumatology; subdivided in weekly working hours dedicated to rheumatic patients, administration, research and teaching. In addition, the following questions were asked: number of patients with RMD cared for per week; percentage of patients with inflammatory and non-inflammatory RMD; amount of time dedicated to first visits of patients; amount of time dedicated to follow-up visits; frequency of follow-up visits for inflammatory and non-inflammatory RMD.

Because the study involved voluntary participation and did not include any further intervention, the Ethics Committee of the sponsoring institution (Medical University of Vienna) issued a waiver obviating the necessity of obtaining informed consent and ethical approval. The survey data was pseudonymized by assigning a unique identifier to each participant. The EULAR points to consider and a current systematic review (7, 8) were followed when the calculation was done.

### Full-Time Equivalent (FTE)

An FTE is a measurement unit to assess the working hours of employed people in a way that makes them comparable, although they may work different numbers of hours a week. Thus, it indicates the workload of an employed person. An FTE in Austria is usually calculated with a workload of 40 h on 5 days per week, on 230 days per year (calculated after deduction of sick leave and holidays). For example, a part-time worker employed for 20 h a week, where full-time work consists of 40 h, is counted as 0.5 FTE. The workforce of a country can then be added up and expressed as the number of full-time equivalents^2^. In our FTE calculation (see results section), direct medical care is defined as a direct contact with the patient (e.g., visits) and rheumatology care combines visits, research and teaching, and administrative work for the patient.

### The German Model

The need for rheumatology services was estimated, using an approach recently published for Germany (9, 10). Because there are currently no estimates for incidence and prevalence exclusively for Austria, German prevalence and incidence rates were used, both countries lying in the same geographic region and having a similar ethic background. Since gout was not included in the German calculations, and the percentage of patients needing to be seen by rheumatologists is unknown, we sent out a questionnaire to all nine members of the gout working group of the Austrian Society of Rheumatology. This was to estimate the percentage of gout patients to be seen by a rheumatologist and in which time intervals.

In the German model, the rheumatologist is responsible for the care of patients with inflammatory RMDs and contributes to the care of patients with severe forms of non-inflammatory RMDs. The prevalence of inflammatory RMDs is estimated to be 2.1%, whereas the incidence is 0.1% in the adult population. Patients with an already diagnosed inflammatory RMD should usually have four follow-up visits per year. The number of individuals suffering from non-inflammatory RMDs, including pain syndromes and osteoporosis, is estimated to be 26,000 per 100,000 adults. At least 10% of these patients should see a rheumatologist once in a year.

The substantial majority of rheumatic patients is managed in an outpatient setting and only a minority of patients are hospitalized. In order to account for this group of patients, we also calculated the number of rheumatologists required to manage the inpatient group. Again, we followed the approach published by the German Society of Rheumatology (9, 10). For hospital care, five beds per 100.000 adult inhabitants are calculated and one Rheumatologist per 10–15 beds is needed. For inpatient and outpatient rehabilitation, 4 beds or rehabilitation facilities per 100.000 adults and one full time rheumatologist for 40 beds are calculated.

The study was conducted from July to October 2017 and is an estimation of the current need in Austria.

The results are divided into a supply and needs section; the latter starts with time allocation for clinical rheumatology visits. This was subsequently used for the need calculation.

### Analysis

Variables showed markedly skewed distributions. Therefore, median and interquartile range (IQR) of variables are presented. Differences between different groups of rheumatologists (categorized according to workplace setting and gender) were compared using Kruskal Wallis test. We performed all the analyses with Stata Statistical Software (Release 13 IC; StataCorp LP).

The study was supported by a dedicated grant from the Austrian Society of Rheumatology and Rehabilitation (ÖGR).

## Results

### Respondents

One hundred and forty-nine of the 215 (=69%) registered specialists for Internal Medicine and rheumatology completed the questionnaire. Of these, 9 stated that they did not work as rheumatologists and were therefore excluded from further analyses, giving a total sample of *n* = 140. Median age was 53 (IQR 46–57). Working time upon completion of rheumatology training was median 11 years (IQR 6–17). Thirty two (25.8%) of the respondents were female (sex was only indicated by 124 participants). Forty three (30.7%) indicated as working solely in a hospital, 38 (27.1%) worked solely in an office. Fifty nine (42.1%) had both hospital and office-based workplaces. Because only a few of the Austrian rheumatologists have a contract with the public/general health insurance companies, the latter were predominantly hospital based, with an additional small private office.

### Supply of Rheumatology Care

#### Weekly Work Hours and Time Allocation to Patient Care, Administration, and Research/Teaching

The weekly working hours indicated by the respondent rheumatologists were median 50 (IQR 45–60) (see Table 1). The average weekly working hours for female rheumatologists were median 45 (IQR 45–51), for male 50 (IQR 41–60). Weekly working hours did not correlate with age (*p* = 0.4051) or sex (*p* = 0.5411). There was no significant difference in frequency in hospital based, office based and both hospital and office based rheumatologists, in neither female nor male rheumatologists (*p* = 0.575). Female rheumatologists are significantly younger (*p* = 0.0192).

**Table 1:** Weekly working hours.

**Setting**	**Weekly working hours**
Hospital based	48 (IQR 40–55)
Office based	50 (IQR 40–55)
Both office and hospital based	50 (IQR 35–60)
All settings	50 (IQR 45–60)

**Figure 1:**
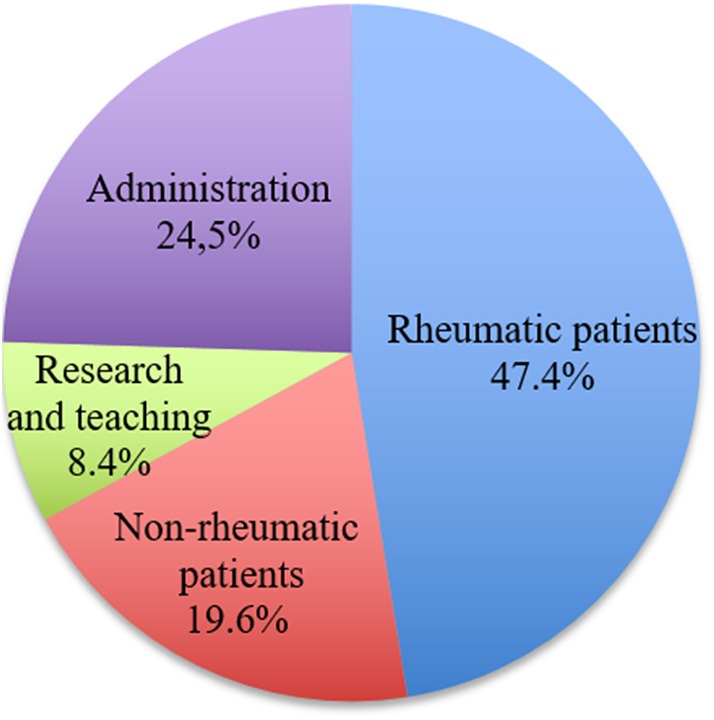
Distribution of working hours per week in percent, all settings (hospital based, office based, both hospital and office based).

Rheumatologists dedicate almost half of their working time to care of patients suffering from RMD (median 24 h per week (IQR 15–30). A median of 10 (IQR 6–15) h are dedicated to administration and a median of 1.5 h (IQR 0–5) are spent on research and teaching. Approximately 20% of the weekly working hours are allocated to care of patients with non-RMDs (patients with general internal conditions) (see also Figure 1).

When comparing rheumatologists working in hospitals only (*n* = 43), in the office only (*n* = 38), and working in both settings (*n* = 59), Table 2 shows the distribution of time dedicated to care of rheumatic patients, non-rheumatic patients, administration, and research and teaching.

**Table 2:** Distribution of working time according to workplace setting.

**Distribution of working time**	**Hospital based (*n* = 43) (%)**	**Office based (*n* = 38) (%)**	**Both hospital and office based (*n* = 59) (%)**	**All settings (*n* = 140) (%)**
Direct medical care for RMD patients	40.9	53.4	48.2	47.4
Direct medical care for non-RMD patients	21.2	22.3	17.2	19.6
Research and teaching	9.4	4.4	10.1	8.4
Administration	28.5	19.9	24.5	24.5

#### Full-Time-Equivalents

In order to estimate the “full-time equivalents” (FTE) available to patient care by the specialists in Internal Medicine and rheumatology, the responses were extrapolated as follows: among the 149 respondents, 9 (6%) did not work as rheumatologists, therefore, we expected the same percentage of “title only” rheumatologists among all 215, leaving an estimate of 202 “true” rheumatologists. Thus, based on the weekly working hours (median 50; see above) and the number of rheumatologists in Austria (202), a total of 10,100 weekly work hours are performed by Austrian rheumatologists. Therefore, 252.5 (10,100:40) FTEs are provided by Austrian rheumatologists. The proportion of time spent on direct medical care for patients with RMDs is 47.4% (4787.4 h). 32.9% of the work hours are spent on research, teaching, and administration (3322.9 h). When one assumes that the rheumatologists spend the same proportion of their time in research, teaching, and administration for RMD and non-RMD patients, another 2350.8 h are spent on “rheumatology care” (which includes administrative work and research/teaching). In total, therefore, Austrian rheumatologists provide 7138.2 weekly hours for RMD-patients. The number of full-time equivalents (FTE; according to Austrian law a full work-week has 40 h) available for rheumatology care thus can be calculated to be 178.5.

Overall, rheumatologists see a median of 40 (IQR 20–50) RMD patients/week.

### Need for Rheumatology Care

#### Outpatient Care

Time allocation for clinical rheumatology visits as indicated by the respondent rheumatologists is shown in Table 3.

**Table 3:** Time allocation for clinical rheumatology visits.

**Time allocation**	**First visit**	**Follow up visit**
Hospital based (*n* = 43)	30 min (IQR 30–45)	15 min (IQR 15–20)
Office based (*n* = 38)	30 min (IQR 35–45)	20 min (IQR 15–30)
Both hospital and office based (*n* = 59)	40 min (IQR 30–45)	20 min (IQR 15–25)
All settings (*n* = 140)	30 min (IQR 30–45)	20 min (IQR 15–25)

The estimated interval for follow up visits for patients with inflammatory rheumatic diseases is median 3 months (IQR 3–4), without any difference in the 3 professional settings (*p* = 0.072). The estimated interval for follow up visits with non-inflammatory rheumatic diseases, for all settings, is median 6 months (IQR 6–10.5). There is no difference between the 3 settings (*p* = 0.31). Thirty one rheumatologists (22%) offer no further follow up for non-inflammatory rheumatic diseases.

The median percentage of patients suffering from non-inflammatory RMDs, cared for by the respondents, is 37.5 4(IQR 20–60) (all settings). Among RMD patients seen by hospital-only based rheumatologists, a median of 30% (IQR 10–40) suffer from non-inflammatory RMDs. For office-only based rheumatologists this figure is 50% (IQR 30–70), and for both hospital and office based rheumatologists 40% (IQR 25–60).

Using an estimated prevalence of 2.1% (10) of inflammatory rheumatic diseases (rheumatoid arthritis, axial spondyloarthritis, psoriatic arthritis, connective tissue diseases, and vasculitides), the number of affected adults in Austria is ~150,000 (10). The annual incidence of inflammatory rheumatic diseases is estimated at 0.1% of the adult population (10).

According to our survey, patients with an already diagnosed inflammatory RMD usually have 4 follow-up visits per year with a rheumatologist. Austrian rheumatologists schedule 20 min for such follow-up visits. Therefore, for every 100,000 adults (assuming 2,100 cases (=2.1%) of established inflammatory rheumatic disease × 4 visits × 20 min), the annual time needed is 2,800 h/year (2,100 × 4 × 20 min = 168,000:60 = 2,800).

For patients with inflammatory disease, with an annual incidence of 0.1%, 50 h (=100 cases at 30 min) per 100,000 adult inhabitants are needed.

In addition, patients with gout (estimated prevalence 1.4%) (11, 12), occasionally need to be seen by rheumatologists, because treatment and surveillance of patients with severe gout has become much more complex in the last couple of years. According to our survey, sent to the members of the gout working group of the ÖGR, we projected that 10% of gout patients see a rheumatologist twice a year (0.14% = 140 per 100,000); an additional 116.7 h/year for 100,000 adults are required (140 × 30 min for first visit + 140 × 20 min for follow up visit = 4,200 + 2,800 = 7,000:60 = 116.67 h).

In addition, for newly referred patients, among whom there is a substantial percentage of individuals with non-inflammatory rheumatic diseases, 30 min are allocated by the surveyed rheumatologists.

With regards to the German Rheumatism Research Center (DRFZ) 26,000 per 100,000 adults suffer from osteoarthritis, chronic pain syndromes, osteoporosis and other non-inflammatory musculoskeletal disorders (9).

We estimate, in line with the German Society of Rheumatology, that at least 10% of these patients, especially with severe non-inflammatory rheumatic disorders, see a rheumatologist once a year, e.g., for treatment recommendations or different diagnostic reasons. Therefore, for every 100,000 inhabitants, 1,300 h (10% of 26, 000 = 2,600 × 30 min = 78,000:60) are necessary.

We calculated a total time needed as 4266.7 rheumatic working hours per 100,000 adults per year (see Table 4). Austrian rheumatologists work, on average, 50 h a week, spending 47.4% of this time (=24 h per week) for the care of patients with rheumatic diseases. That is 4.8 rheumatic working hours per day (24 working hours in 5 days = 24:5 = 4.8), working on 230 days per year, which results in 1,104 working hours per year (4.8 × 230). Consequentially, the need per 100,000 adult inhabitants per year (4266.7 h/1,104 h): = 3.86 rheumatologists per 100,000 adults.

**Table 4:** Time need per 100,000 adult inhabitants per year.

	**Working hours**
Prevalent inflammatory disorders	2,800 h
Incident inflammatory disorders	50 h
Gout	116.7 h
Severe non-inflammatory disorders	1,300 h
Total	4266.7 h

Calculated for Austria^3^, with 7.03 million adult inhabitants, there is a need for 271.36 rheumatologists for outpatient rheumatology care (3.86 × 70.3 = 271.36).

This results in FTEs [calculated with a workload of 40 h, instead of the 50 h actual working time according to our survey, and based on 230 days per year (see methods section)], working 18.96 h per week and 872.16 h per year (18.96:5 × 230) for care of RMD patients Consequently, the need per 100,000 adult inhabitants is 4.89 FTEs (4266.7:872.16). Calculated for Austria there is a need of 343 FTEs (4.89 × 70.3) for outpatient care.

#### Inpatient and Rehabilitative Rheumatic Care

The German Society of Rheumatology calculates a need for 5 beds/100,000 adults for hospital treatment (9) and a need for acute inpatient care of 1 rheumatologist per 10–15 beds, resulting in 0.33 rheumatologists per 100,000 adult inhabitants.

Therefore, in Austria, there is a need for 351.5 beds (5 × 70.3) and a request for 23.4 additional rheumatologists (351.5:15; low estimate).

The German Society of Rheumatology calculates a need for 4 beds, or outpatient rehabilitation facilities, per 100,000 adults for rehabilitative care (9) and a need for 1 rheumatologist per 40 beds, or outpatient rehabilitation facilities. This results in 0.1 rheumatologists per 100,000 rehabilitative patients.

For Austria, with 7.03 million adults, there is a need for 281.2 (70.3 × 4) beds, or inpatient rehabilitation facilities, for rehabilitative care and a need for an additional 7.03 rheumatologists (281.2:40) in rehabilitative care.

Thus, there is a total need for 4.29 rheumatologists per 100.000 (3.86 + 0.33 + 0.1 = 4.29) for outpatient care, and inpatient and rehabilitative rheumatology care.

For 30.43 rheumatologists for inpatient and rehabilitative care according to the German model, we calculated 41.04 FTE (30.43 × 54[weekly workload in Germany]:40).

#### Total Need for Rheumatologists in Austria

Calculated for Austria^3^, inclusive of outpatient, inpatient and rehabilitative rheumatological care, with 70.3 million adult inhabitants, there is a total need for 301.79 rheumatologists (271.36 + 23.4 + 7.03), which is in stark contrast to the existing workforce (see Table 5).

**Table 5:** Total need of rheumatologists calculated for Austria with 7.03 million adult inhabitants.

	**Need for rheumatologists in Austria**
Outpatient rheumatic care (including gout)	271.36
Inpatient rheumatic care	23.4
Rehabilitative rheumatic care	7.03
Total	301.79

#### Alternative Scenarios

Scenario 1: An alternative solution would be to increase the percentage of time dedicated to RMD care: e.g., a rheumatologist would see solely RMD patients (and not spend 19.6% of his working-time for non-RMD care). Instead of 47.4% (=24 h per week) a rheumatologist would use 67% of his working-time for the care of patients with rheumatic diseases (47.4% + 19.6% = 67%). That is 33.5 h per week, 6.7 h with RMD patients per day (33.5:5 = 6.7) and 1,541 working-hours per year (6.7 × 230). In this scenario, the need per 100,000 adult inhabitants would be 2.76 rheumatologists per 100.000 (4266.7:1,541) for outpatient care.

The total need would be 3.19 (2.76 + 0.33 + 0.1) rheumatologists per 100.000 (outpatient care and inpatient and rehabilitative rheumatology care). Calculated for Austria, with 7.03 million adult inhabitants (9), this converts to 224.25 rheumatologists (3.19 × 70.3 = 224.25) according to the actual work hours in our survey.

Expressed in FTEs [67% of a working time of 40 h per week = 26.8 h per week for care of RMD patients and 1232.8 h per year (26.8:5 × 230)], the need per 100.000 adult inhabitants would be 3.70 FTEs (4266.7:1232.8). Calculated for Austria, there would be a need for 260 FTEs (3.70 × 70.3).

Scenario 2: If you calculate only 10% of a rheumatologists' working-time dedicated to non-RMD patients, an Austrian rheumatologist would spend 57% (47.4% + 9.6%) of his working time with RMD patients. That is 28.5 h per week, 5.7 h per day (28.5:5 = 5.7) and 1,311 h per year (5.7 × 230) for outpatient care. In this scenario, the need per 100,000 adults would be 3.25 rheumatologists per 100,000 inhabitants (4266.7/1,311) according to the actual work hours. The total need would be 3.68 (3.25 + 0.33 + 0.1) rheumatologists per 100.000 (outpatient care and inpatient and rehabilitative rheumatology care). Calculated for Austria, this results in a need for 258.70 rheumatologists (3.68 × 70.3 = 258.70).

Expressed in FTEs [57% of a working time of 40 h per week = 22.8 h per week for care of RMD patients and 1048.8 h per year (22.8:5 × 230)], the need per 100.000 adult inhabitants would be 4.06 FTEs (4266.7:1048.8). Calculated for Austria, there would be a need for 285.4 FTEs (4.06 × 70.3).

## Discussion

Our workforce study, with the participation of almost 70 percent of all Austrian rheumatologists, demonstrates a supply of 178.5 FTEs available for care of adult patients with RMDs. This supply is provided by ~200 doctors/specialists in Internal Medicine and rheumatology. Our investigation was the first ever study of its kind in Austria, including all working rheumatologists registered with the Austrian Medical Association, which is mandatory to be allowed to practice Medicine. The strengths of our study are the high response rate and the use of a meticulously crafted questionnaire, to adequately capture time intervals, in order to avoid recall bias.

The study resulted, given the particular structure of the Austrian health care system and the time allocation for direct patient care resulting from our survey, in the need for 4.29 rheumatologists per 100,000 adult inhabitants for rheumatology care. Correspondingly, 301.79 rheumatologists are needed to fully serve the Austrian adult population. In contrast, in a “perfect world scenario,” where rheumatologists only see RMD patients, there would be a need for 3.19 rheumatologists per 100,000 adult inhabitants for rheumatology care. Under such circumstances, 224.45 rheumatologists would be needed to fully serve the Austrian adult population. However, it is doubtful, whether such a “perfect world scenario” is realistically achievable.

Results from the literature have shown that there are only a few scientific publications dealing with workforce in rheumatology thus far (1, 6, 8). A current review from Dejaco et al. (1) about workforce planning in Western countries, included 14 scientific publications from 5 different countries. Research was heterogeneous concerning methods, investigated time periods and variables used for the calculation of manpower requirements. Therefore, there are different estimations of rheumatology workforce needs, between 0.7 [UK, calculated for 1988 (13)] up to 3.5 [Spain, calculated for 2021 (14)] rheumatologists. Most of the published models are in the range of 2 rheumatologists per 100,000 [Germany, calculated for 2008 (15)]. Our estimate which is based on “real-world”-data, is considerably higher than the estimates in the published literature. One explanation may be that the calculations so far are based on assumptions and/or estimates of time demands based on theoretical considerations. (Not only) Austrian rheumatologists, whether office- or hospital-based, are responsible, due to their training, for treating RMD patients with sometimes numerous comorbidities, which demand for additional consultation time. Predominantly in hospitals, rheumatologists often work in general internal units and have to take care of other internal diseases as well. This would explain that almost 20% of their work is scheduled with non-RMD patients.

The setting of our study is in one country and within the framework of the Austrian Health Service, which allows free access to a physician of choice for every patient and has a universal health insurance coverage. Furthermore, there is a long tradition of rehabilitation services, which offer patients with chronic disease, plus patients after surgery or traumas, either inpatient or outpatient rehabilitative care.

Our study has some limitations. It is a nationwide survey limited to Austrian clinical practice, which, as reflected in the comparison with German data may differ, in some parts, from practice in other countries. In contrast to Germany, we calculated 30 min (instead of 15 min) for a visit with a severe non-inflammatory rheumatic disorder. On the whole, results of comparisons between different health care systems have to be interpreted with caution. Another limitation is that rheumatologist's answers to questions on practice patterns may not be supported by clinical documentation. That is to say, some information regarding time periods, of both first and follow up visits, might be estimated and not precise. Potential biases, such as wrong or imprecise estimates by the respondents, have to be considered. However, health workforce planning is not an exact quantification science. Several calculations were based on expert opinion, because evidence was limited or absent for many aspects (7).

One additional imprecision may stem from the fact that we assumed the same proportion of time was spent for research and teaching, for both RMD- and non-RMD-patients. However, even if no research and teaching time was dedicated to non-RMD-patients, leaving “their” 8% share only for RMD patients, this would not have changed the results and/or conclusions substantially.

A projection of optimized specialist care for patients with musculoskeletal disorders has several impacts. It depends on retirement, the next generation of doctors with a different attitude to work-life balance and on demography, in terms of a future population development and the prevalence of rheumatic diseases, considering an aging society (1, 6). New treatment recommendations, the implementation of a “treat to target” (T2T) strategy in clinical practice, as well as the monitoring of more complex therapies such as biologicals, lead to more specialist follow up visits and a more stringent surveillance than in years gone by (2–4). Needs significantly outweigh supply in rheumatologists. Due to this shortage in Austria, people with inflammatory RMDs may occasionally be seen by disciplines other than rheumatologists, such as GPs or general internal specialists.

The 2015 American College of Rheumatology workforce study projects a significant adult rheumatology workforce shortage over the next 15 years in the United States (6). Current and future Europe-wide developments with regards to a maximum working time of 48 h (night calls included) have to be considered (1). We have to consider that in the future there will be a change in gender distribution of rheumatologists. Even today, female rheumatologists are significantly younger than men. In addition, incorporation of Health professionals (HP) in medical information, treatment and surveillance of patients with inflammatory rheumatic diseases will improve rheumatology care and, indirectly, possibly reduce the need for rheumatologists. There is data to show that, at least in gout, nurses can be much better at providing T2T (4) treatment than GPs (16). Most of the rheumatology units work already together with qualified HPs. Currently rheumatology specialty training for HPs in Vienna and Western Austria is offered by the professional society of HPs^4^.

We have to consider regional heterogeneity of supply and needs, which may vary across countries, regions, as well as rural vs. urban areas (6, 7). All these aspects may have a substantial effect on future developments and health care planning (6), especially with large urban areas growing in population and rural areas losing inhabitants.

We do not know who, in the future, will take care of patients with osteoporosis, chronic pain syndromes, and non-inflammatory rheumatic diseases. Currently, these patients are treated by their family doctors, orthopedists, neurologists, and rheumatologists (1). Will the spectrum of diseases and the proportion of patients for rheumatology care expand in the future? There are a lot of aspects to be considered, which aggravate rheumatology workforce planning. In addition, approximately half of the rheumatologists will reach retirement age within the next 15 years, in Austria. Therefore, the discipline faces possible shortages to replace the retiring colleagues, considering the fact that only ~50 training posts are available nationwide (meaning that only 8 rheumatologists/year are going to finish training).

## Summary

Our model integrates supply and needs for a respective country and presents results as full-time equivalents and numbers of rheumatologists (7). Given the particular structure of the Austrian health care system, our calculation results in a need for 4.29 rheumatologists per 100,000 adults (outpatient, inpatient, and rehabilitative care). This is considerably higher compared to workforce needs in previous research (1, 13–15). It could further increase, if we put aspects like a reduction of working hours in the European Union, or a larger range of diseases to be treated by rheumatologists, into account. However, the results of our study are a conservative estimate (as mentioned above) with a powerful policy impact on healthcare workforce planning.

## Data Availability Statement

The datasets generated for this study are available on request to the corresponding author.

## Author Contributions

RP, AV, and KM: conception and design. RP, AV, RH, and KM: acquisition of data. RH and HP: statistical analysis. RP, AV, HP, and KM: manuscript preparation.

### Conflict of Interest

AV was employed by the company Academy of Value in Health GmbH. The remaining authors declare that the research was conducted in the absence of any commercial or financial relationships that could be construed as a potential conflict of interest.

## Footnotes

^1^https://ec.europa.eu/health//sites/health/files/workforce/docs/health_workforce_study_2012_report_en.pdf

^2^http://epp.eurostat.ec.europa.eu/statistics_explained/index.php/Glossary:Full-time_equivalent_(FTE) (last downloaded 8.12.2019)

^3^http://www.statistik-austria.at. Population statistic 2016.

^4^https://www.rheumatologie.at/gesundheitsberufe/fortbildung_weiterbildung/ (last downloaded 16.12.2019).
